# Therapeutic Modulation of the Nox2–Hv1–ROS Axis by Botulinum Neurotoxin A Confers Protection Against CoCl_2_-Induced Retinal Hypoxic Injury

**DOI:** 10.3390/ijms262110806

**Published:** 2025-11-06

**Authors:** Hey Jin Lee, Mira Park, Hyun-Ah Shin, Helen Lew

**Affiliations:** 1 CHA R&D Institute, CHA University, Seongnam 13496, Gyeonggi-do, Republic of Korea; 4378nm@chamc.co.kr; 2 Department of Ophthalmology, Bundang CHA Medical Center, CHA University, Seongnam 13496, Gyeonggi-do, Republic of Korea; hoohoo99@chamc.co.kr; 3 Department of Biomedical Science, CHA University, Seongnam 13496, Gyeonggi-do, Republic of Korea; sha9547@naver.com

**Keywords:** Botulinum Toxin A, Hv1, hypoxia, Nox2, oxidative stress, retinal ganglion cells

## Abstract

Neuroinflammation and oxidative stress are key drivers of various ocular diseases. Experimental hypoxia, modeled using cobalt chloride (CoCl_2_), induces hypoxia-inducible factor 1-alpha (HIF-1α) stabilization, mitochondrial dysfunction, and excessive reactive oxygen species (ROS) production, primarily via the NADPH oxidase 2 (Nox2)–voltage-gated proton channel Hv1 axis. Although Botulinum neurotoxin type A (BoNT/A) is classically recognized for SNAP-25 cleavage, recent studies suggest broader anti-inflammatory and neuroprotective effects. We evaluated BoNT/A in R28 retinal precursor cells and ex vivo retinal explants subjected to CoCl_2_-induced hypoxic stress. BoNT/A pretreatment attenuated CoCl_2_-induced upregulation of HIF-1α, Hv1, Nox2, NOD-like receptor protein 3 (NLRP3), COX2, and nuclear factor kappa B (NF-κB), while enhancing protective mediators including suppressor of cytokine signaling 3 (SOCS3), Growth Associated Protein 43 (Gap43), and Syntaxin12. Brn3a expression and retinal architecture were preserved, apoptotic cell death reduced, and glial activation suppressed. Moreover, BoNT/A decreased mitochondrial ROS accumulation, restored voltage-dependent anion channel 1 (VDAC1) distribution, and partially stabilized intracellular pH. These findings indicate that BoNT/A mitigates oxidative stress and inflammation in hypoxia-driven retinal injury, at least in part, via modulation of the Nox2–Hv1–ROS axis, and support its potential as a therapeutic candidate for ocular disorders associated with hypoxia and neuroinflammation.

## 1. Introduction

Globally, more than 2.2 billion people are estimated to have some form of vision impairment, and roughly 1 billion cases are considered avoidable or unmet (Blindness and vision impairment–WHO fact sheet). In the Asia–Pacific region, visual impairment remains a major public health issue with substantial regional variation. Previously, Tang et al. reported the prevalence of blindness was 1.0% using the WHO criteria. A recent nationwide study reported the overall prevalence of blindness was 0.473% among Korean adults [1].

Neuroinflammation plays a pivotal role in the pathogenesis of vision-threatening disorders, such as age-related macular degeneration, diabetic retinopathy, glaucoma, and optic neuropathy [2,3,4,5]. Notably, regional data indicate that the prevalence of glaucoma in East Asia, including South Korea, ranges from 2.1% to 3.3% among adults older than 40 years [6,7]. Approximately 250,000 individuals were registered as visually impaired in 2021, underscoring the substantial national burden of vision-threatening eye disease in South Korea [8].

In response to stress signals, retinal microglia and astrocytes—the primary resident immune cells—become activated and release proinflammatory mediators that propagate neurodegenerative cascades [9,10,11,12]. Excessive generation of reactive oxygen species (ROS), primarily driven by the NADPH oxidase (NOX) enzyme family, is a major contributor to this process, with Nox2 representing the predominant isoform in microglia [13,14]. Optimal Nox2 activity depends on proton efflux mediated by the voltage-gated proton channel Hv1, which maintains charge balance and intracellular pH during respiratory burst activity [15,16]. Consequently, dysregulation of the Hv1–Nox2 axis is a critical mechanism driving oxidative stress and neuroinflammation in retinal and other neurodegenerative diseases [17,18,19].

To replicate hypoxic stress experimentally in retinal systems, cobalt chloride (CoCl_2_) is commonly used as a hypoxia-mimetic agent. CoCl_2_ stabilizes hypoxia-inducible factor 1-alpha (HIF-1α) by inhibiting prolyl hydroxylases, preventing HIF-1α degradation under normoxic conditions [20,21,22]. Stabilized HIF-1α translocates to the nucleus, where it promotes the transcription of hypoxia-responsive genes and disrupts mitochondrial homeostasis [23,24]. This disruption enhances mitochondrial ROS generation and further activates Nox2, intensifying oxidative stress [25,26,27]. Mitochondria are central to this interaction, as hypoxia-induced metabolic alterations modify redox signaling, ROS production, and inflammasome activation [28,29]. Among mitochondrial components, voltage-dependent anion channel 1 (VDAC1)—a key outer membrane protein—integrates oxidative stress and apoptotic signaling; its oligomerization has been associated with NOD-like receptor protein 3 (NLRP3) inflammasome activation under toxic stress conditions [30,31]. Collectively, these mechanisms emphasize the intricate crosstalk between hypoxia, mitochondrial dysfunction, and inflammatory signaling that drives retinal degeneration.

In this context, R28 retinal precursor cells and ex vivo retinal explant cultures serve as complementary platforms for investigating hypoxia- and inflammation-induced retinal injury. R28 cells, derived from neonatal rat retina, retain key characteristics of retinal neuronal progenitors and are used to study hypoxia-mediated injury, oxidative stress, and neuroprotective mechanisms [32,33,34]. In parallel, retinal explant cultures preserve the native laminar organization and intercellular interactions of the retina, providing a physiologically relevant ex vivo system for examining inflammatory and degenerative responses [35,36,37]. Notably, prior studies using diabetic and retinopathy of prematurity models have demonstrated that blood–retinal barrier disruption and inner retinal neuronal alterations are closely associated with oxidative stress and inflammation, further validating these ex vivo systems for mechanistic investigations [38,39].

Emerging therapeutic strategies are aimed at suppressing Hv1–Nox2-mediated ROS generation to alleviate retinal neuroinflammation. Of these, botulinum neurotoxin type A (BoNT/A), classically known for cleaving SNAP-25 and inhibiting synaptic vesicle exocytosis, has attracted growing interest for its broader immunomodulatory and antioxidant properties [40,41,42,43]. Beyond its local synaptic effects, BoNT/A exhibits long-range retrograde actions within neuronal networks, indicating potential systemic neuroprotective effects. For instance, BoNT/A treatment reduces NOX-dependent ROS production in ischemia–reperfusion models and decreases systemic oxidative stress markers in patients with chronic migraine [44,45,46]. Furthermore, BoNT/A has been reported to attenuate inflammatory signaling in microglial BV-2 cells, suppress NLRP3 inflammasome activation in trigeminal ganglion neurons, and inhibit microglial pyroptosis [47,48,49].

Mechanistically, BoNT/A is now recognized to modulate cytokine signaling in addition to its established effects on synaptic transmission and glial activity. For instance, BoNT/A-induced upregulation of suppressor of cytokine signaling 3 (SOCS3) has been shown to attenuate nuclear factor kappa B (NF-κB)-mediated inflammatory responses [50,51,52]. In ocular contexts, SOCS3 activation has been associated with reduced glial reactivity and inhibition of pathological angiogenesis [51,53,54]. These findings indicate that BoNT/A acts at multiple nodes within inflammatory pathways—including NF-κB, NLRP3, and SOCS3—to mediate its neuroprotective effects.

Although direct evidence connecting BoNT/A to Hv1–Nox2 regulation remains limited, its capacity to suppress upstream inflammatory mediators, alleviate mitochondrial dysfunction, and modulate microglial activity indicates that BoNT/A may represent a promising therapeutic modulator for hypoxia- and inflammation-induced retinal injury [47,50,51]. For example, glaucoma alone affects an estimated 76 million individuals worldwide in 2020 and is projected to rise to 111 million by 2040 [55].

Therefore, the present study aimed to investigate whether BoNT/A confers neuroprotective and anti-inflammatory effects against hypoxia-induced retinal injury by modulating the Hv1–Nox2–ROS axis. We hypothesized that BoNT/A suppresses microglial activation and oxidative stress by inhibiting the Hv1–Nox2 complex while simultaneously promoting protective signaling pathways involving SOCS3, GAP43, and Syntaxin12. Through both in vitro (R28 retinal precursor cells) and ex vivo (rat retinal explant) models, this study sought to elucidate the dual role of BoNT/A in attenuating oxidative neuroinflammation and enhancing neuroprotective responses under hypoxic conditions.

## 2. Results

### 2.1. BoNT/A Regulates Hv1–Nox2-Dependent ROS Generation and Mitochondrial Dynamics to Counteract Hypoxia-Induced Injury in R28 Cells

**Figure 1:**
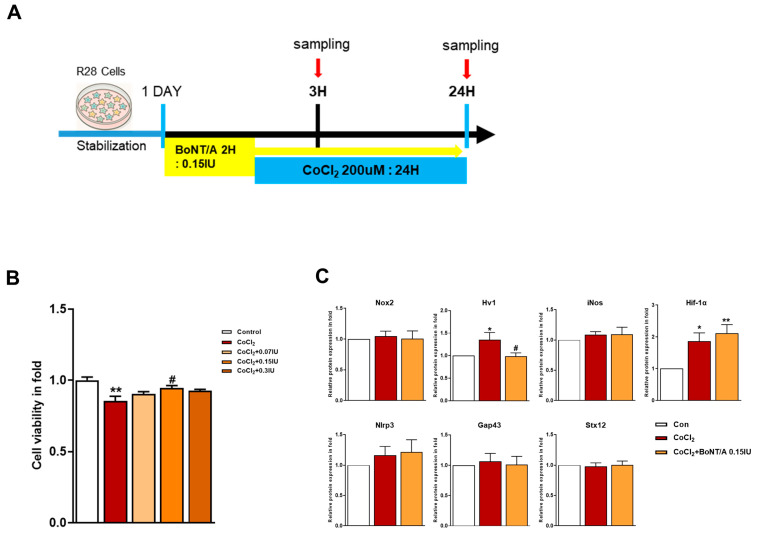

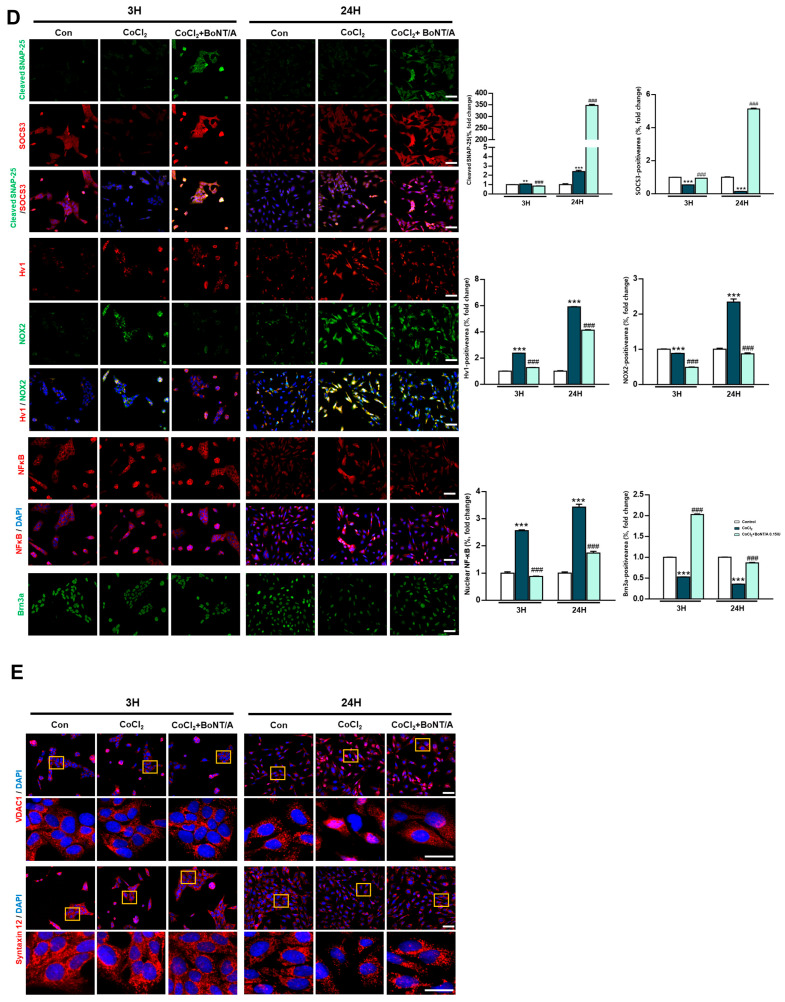
Protective effects of BoNT/A in R28 cells under hypoxic stress. (**A**) Schematic illustration of the experimental timeline. R28 cells were pretreated with BoNT/A for 2 h, followed by CoCl_2_ (200 μM) exposure, and samples were collected at 3 h and 24 h. (**B**) Cell viability was tested using the CCK-8 assay. Data are presented as mean ± SEM (** *p* < 0.01 vs. control; # *p* < 0.05 vs. CoCl_2_). (**C**) Expression levels of target proteins in R28 cells exposed to CoCl_2_ for 3 h. (**D**) Immunocytochemistry analyses revealed multiple BoNT/A-related effects. The first panel confirmed BoNT/A activity by cleaved-SNAP25 staining (green). The second panel showed increased SOCS3 expression after BoNT/A treatment. The third panel demonstrated that BoNT/A suppressed the CoCl_2_-induced upregulation of Hv1 and Nox2. The fourth panel revealed that CoCl_2_ promoted NF-κB nuclear translocation, which was reduced by BoNT/A pretreatment. The fifth panel showed that Brn3a expression, markedly decreased in CoCl_2_-treated cells, was preserved in the CoCl_2_ + BoNT/A group, indicating protection of retinal ganglion cell integrity. Scale bars: 50 μm. (**E**) VDAC1 (red) displayed pronounced perinuclear clustering in CoCl_2_-treated cells, consistent with mitochondrial aggregation under stress, whereas this effect was attenuated by BoNT/A pretreatment (arrows). Syntaxin12 (red), normally extended in filamentous structures, exhibited fragmentation in CoCl_2_ + BoNT/A samples at 24 h, suggesting cleavage or disruption of its morphology. The yellow boxes indicate the regions that were magnified and shown below to highlight these structural changes. Scale bars: 50 μm (overview); 25 μm (insets). Statistical significance was indicated as * *p* < 0.05, ** *p* < 0.01, *** *p* < 0.001 vs. control; # *p* < 0.05, ### *p* < 0.001 vs. CoCl_2_.

To investigate the therapeutic potential of BoNT/A in alleviating hypoxia-induced stress, R28 cells were pretreated with BoNT/A following or concurrent with CoCl_2_ exposure (Figure 1A). Cytotoxicity assays confirmed that BoNT/A was non-toxic at the tested concentrations, supporting its suitability for subsequent analyses (Figure 1B and Supplementary Table S1). A quantitative summary of cell viability following CoCl_2_-induced hypoxic stress and BoNT/A treatment is provided in Supplementary Table S1). Western blot evaluation of Nox2, Hv1, inducible nitric oxide synthase (iNOS), HIF-1α, and NLRP3 expression in CoCl_2_-treated R28 cells revealed that BoNT/A treatment significantly reduced Hv1 expression relative to the CoCl_2_ group, suggesting that BoNT/A ameliorates Hv1–Nox2 activation under hypoxic conditions rather than preventing its initiation. In contrast, GAP43 and Syntaxin12 exhibited significant alterations at the 24 h time point, implying that their regulation may occur during the recovery phase of injury progression or recovery (Figure 1C).

Immunocytochemical analyses corroborated these findings (Figure 1D). Cleaved-SNAP25 staining confirmed the enzymatic activity of BoNT/A, whereas SOCS3 expression was significantly upregulated in BoNT/A-treated cells. BoNT/A treatment alleviated CoCl_2_-induced upregulation of Hv1 and Nox2 and reduced NF-κB nuclear translocation, reflecting attenuation of inflammatory signaling. Furthermore, Brn3a staining demonstrated restoration of retinal ganglion cell (RGC) markers in the CoCl_2_ + BoNT/A group compared to CoCl_2_ alone, indicating that BoNT/A mitigates hypoxia-induced neuronal damage and supports structural recovery of RGCs.

Mitochondrial stress responses were evaluated using VDAC1 and Syntaxin12 as markers. CoCl_2_ exposure induced prominent perinuclear clustering of VDAC1-positive mitochondria, consistent with stress-induced mitochondrial aggregation (Figure 1E, arrow). In contrast, BoNT/A treatment attenuated this abnormal clustering, suggesting improvement of mitochondrial organization and reduction in localized ROS accumulation in the perinuclear region.

Syntaxin12, typically exhibiting a filamentous pattern, appeared fragmented following CoCl_2_ + BoNT/A treatment at 24 h. Dual immunostaining for cleaved-SNAP25 and Syntaxin12 revealed elongated Syntaxin12 filaments at 3 h but fragmented structures at 24 h, suggesting that BoNT/A may cleave SNAP25 and Syntaxin12 (Figure S1). Quantitative immunocytochemical analyses demonstrated significant group-wise differences for all evaluated markers. Collectively, these findings indicate that BoNT/A alleviates hypoxia-induced oxidative stress and inflammatory responses and promotes partial restoration of neuronal integrity, acting as a therapeutic modulator rather than a solely preventive agent.

### 2.2. BoNT/A Confers Dual Protection Against Oxidative Stress and Apoptosis Under Hypoxia

**Figure 2:**
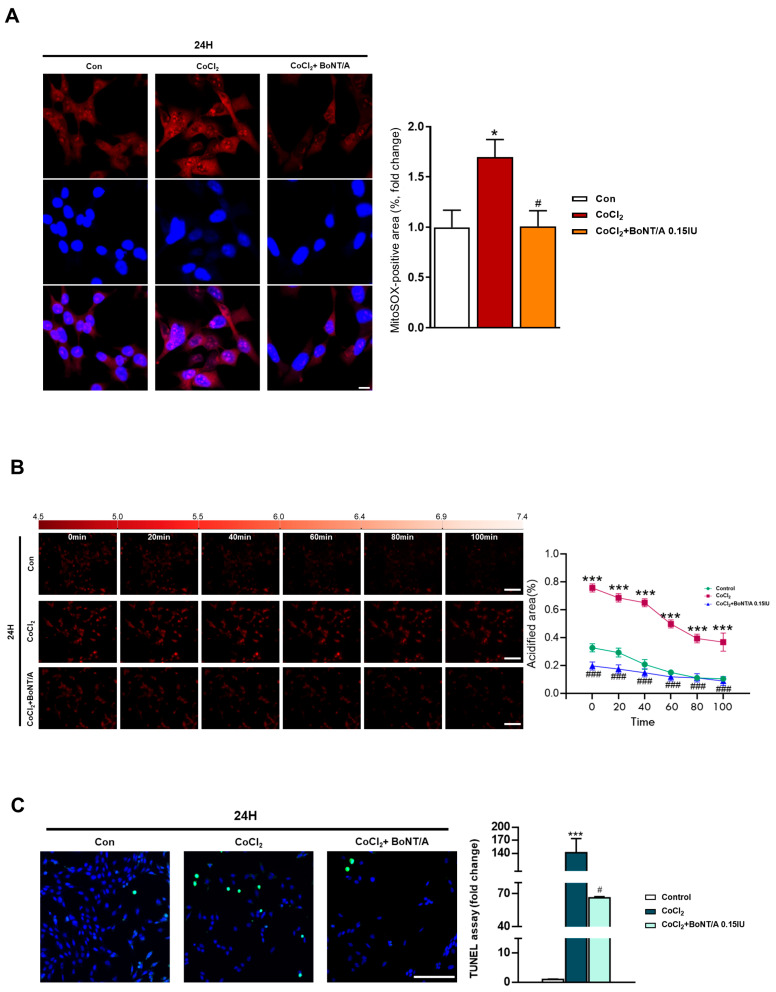
Effects of BoNT/A on mitochondrial ROS production and apoptosis in R28 cells under hypoxia. (**A**) Mitochondrial ROS production was assessed by MitoSOX staining in R28 cells. Representative confocal images from the final time point are shown. Quantitative fluorescence analysis demonstrated that CoCl_2_ (200 μM) markedly increased mitochondrial ROS levels compared with controls, whereas BoNT/A pretreatment (0.15 IU, 2 h) significantly attenuated this elevation. Scale bar: 10 μm. (**B**) Real-time measurement of intracellular pH using the pHrodo™ AM probe. At 3 h, fluorescence intensities were comparable among the Control, CoCl_2_, and CoCl_2_ + BoNT/A groups. By 24 h, CoCl_2_-treated cells exhibited markedly increased red fluorescence, indicating intracellular acidification and pH homeostasis disruption under hypoxic stress. BoNT/A pretreatment partially alleviated these alterations. (**C**) TUNEL assay images of R28 cells under Control, CoCl_2_, and CoCl_2_ + BoNT/A (0.15 IU, 2 h) conditions. Quantitative analysis revealed that CoCl_2_ markedly increased apoptotic nuclei, whereas BoNT/A pretreatment significantly reduced apoptosis, confirming protection against hypoxia-induced cell death. Statistical significance was indicated as * *p* < 0.05, *** *p* < 0.001 vs. control; # *p* < 0.05, ### *p* < 0.001 vs. CoCl_2_. Scale bar: 100 μm.

To investigate the therapeutic effects of BoNT/A in alleviating hypoxic conditions, mitochondrial ROS production and apoptosis were assessed in R28 cells (Figure 2A). Live-cell imaging with MitoSOX revealed that CoCl_2_ treatment markedly increased mitochondrial ROS levels compared with controls, consistent with its hypoxia-mimicking properties (Figure S2). We next examined the effect of BoNT/A on intracellular pH regulation using the pHrodo™ AM probe (Figure 2B). At 3 h, fluorescence intensities were comparable among the Control, CoCl_2_, and CoCl_2_ + BoNT/A groups. By 24 h, CoCl_2_-treated cells exhibited a pronounced increase in red fluorescence intensity, indicating intracellular acidification and disruption of pH homeostasis under hypoxic conditions. BoNT/A treatment attenuated these alterations and partially restored pH balance, suggesting a corrective effect on cellular acid–base homeostasis during hypoxic stress.

Concurrently, terminal deoxynucleotidyl transferase dUTP nick end labeling (TUNEL) assays demonstrated a substantial increase in apoptotic nuclei following CoCl_2_ exposure, with TUNEL-positive cells rising approximately 146-fold compared with controls. BoNT/A treatment significantly reduced this apoptosis by approximately 53%, indicating a strong therapeutic effect in limiting hypoxia-induced cell death (Figure 2C). Quantitative analyses of MitoSOX and TUNEL assays confirmed that BoNT/A ameliorates excessive ROS accumulation and apoptotic signaling. Collectively, these findings demonstrate that BoNT/A acts as a therapeutic modulator that alleviates hypoxia-induced oxidative stress, restores intracellular pH stability, and mitigates apoptotic cell loss, thereby facilitating cellular recovery rather than merely preventing injury progression.

### 2.3. BoNT/A Offers Structural Protection and Reduces Hypoxia-Induced Cell Loss in Ex Vivo Retinal Models

**Figure 3:**
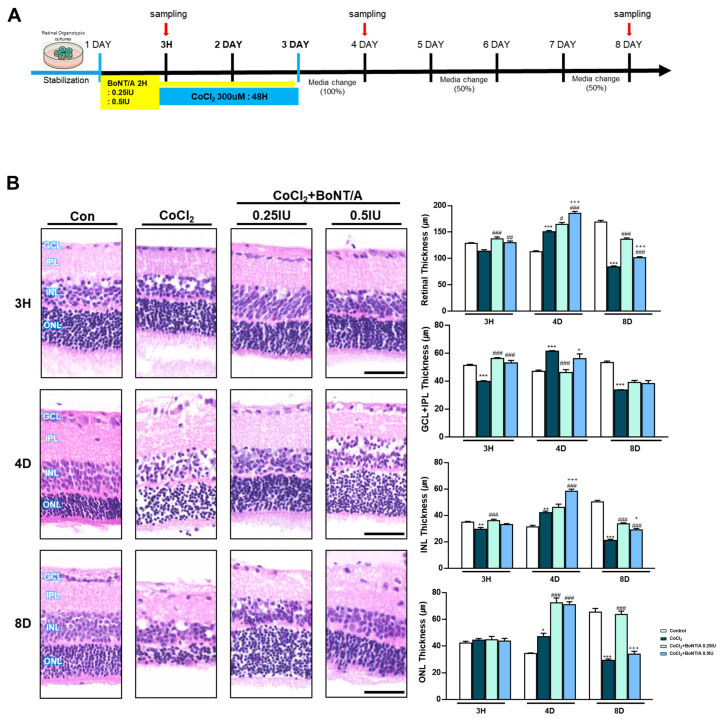

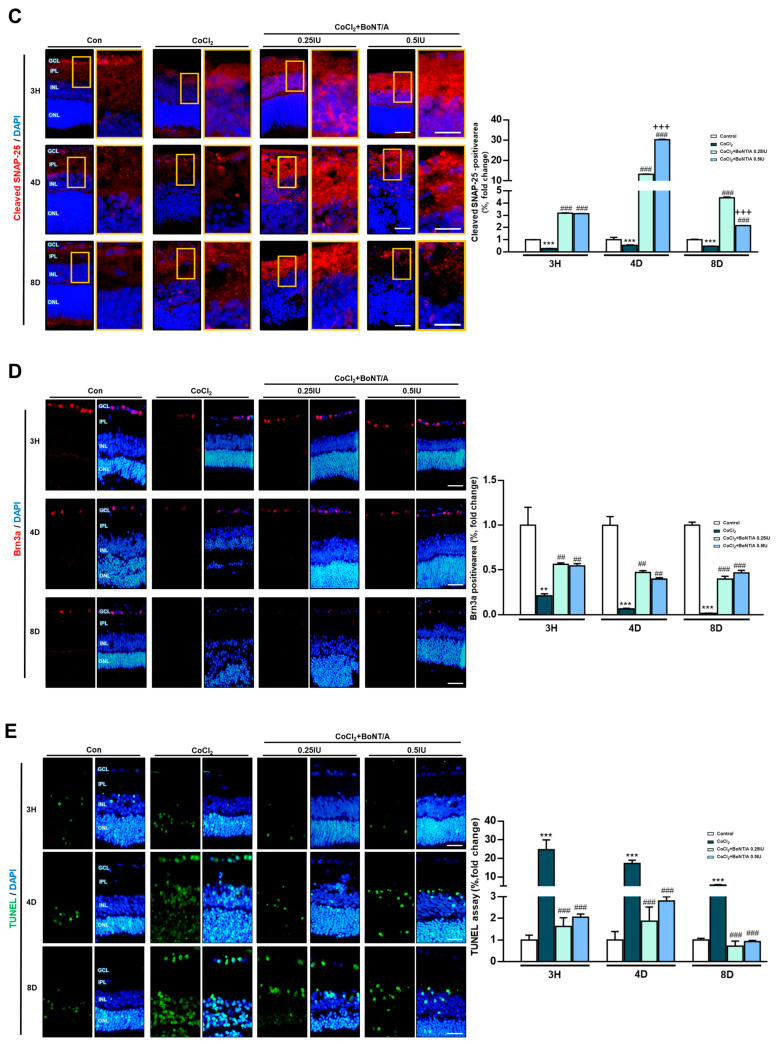

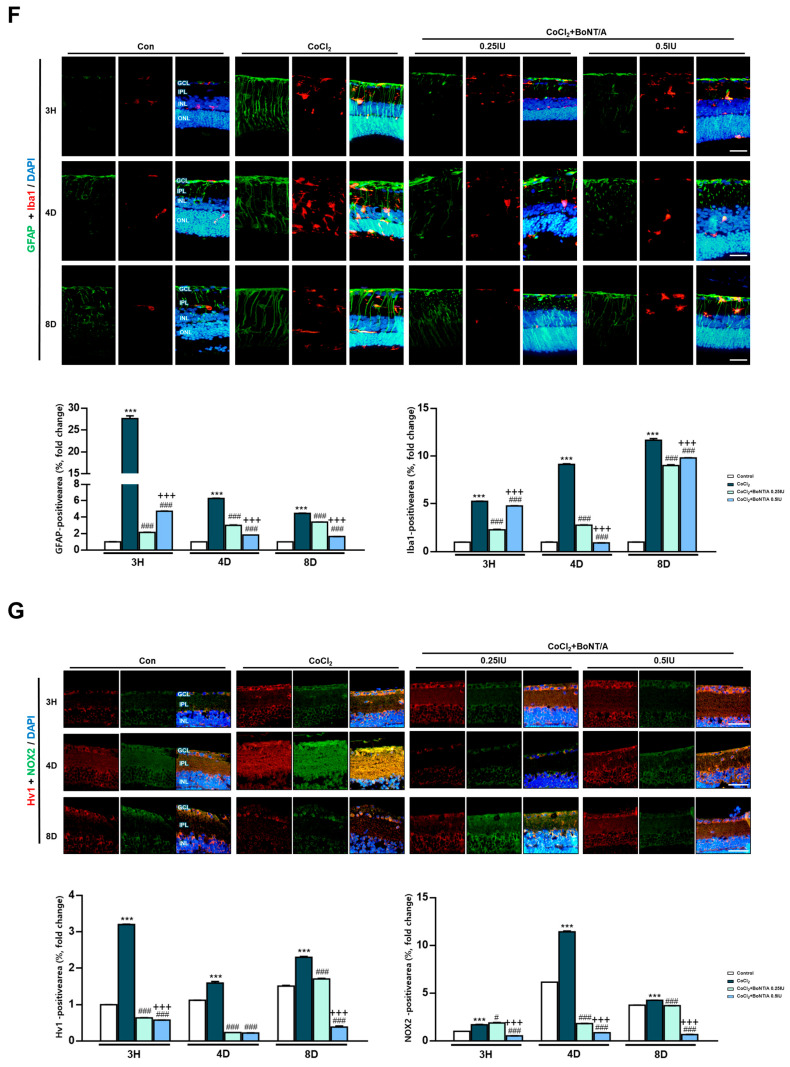

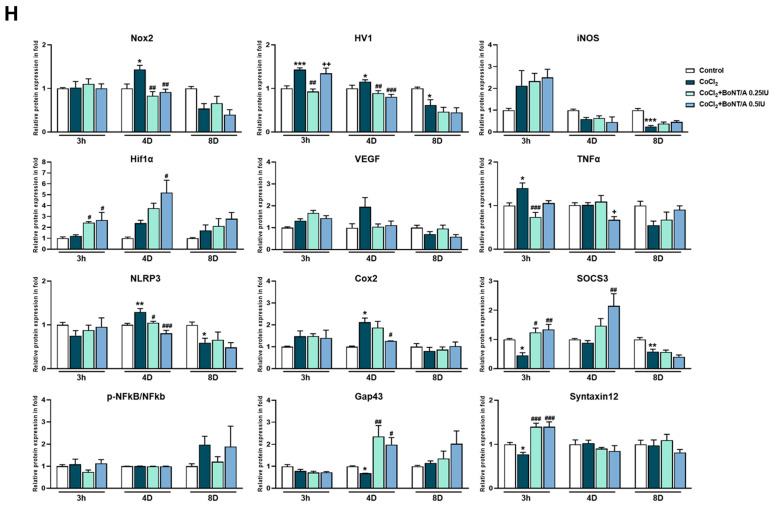
BoNT/A protects retinal structure and reduces retinal ganglion cell loss in an ex vivo model. (**A**) Experimental scheme of the ex vivo retina model. Rat retinas were cultured ex vivo, pretreated with BoNT/A (2 h), and subsequently exposed to CoCl_2_ (300 μM). Samples were collected at 3h, 4D (days), and 8D. (**B**) Representative hematoxylin–eosin (H&E) staining images of paraffin-embedded retinal cross-sections at the indicated time points. Retinal thickness was quantified for the whole retina as well as for specific layers, including the inner plexiform layer (IPL), inner nuclear layer (INL), and outer nuclear layer (ONL). CoCl_2_ treatment caused progressive thinning of the retina and individual layers, whereas BoNT/A pretreatment preserved retinal thickness at levels comparable to controls. Scale bar: 50 μm. (**C**) Immunofluorescence staining of cleaved-SNAP25 confirmed BoNT/A enzymatic activity in retinal tissues. Robust cleaved-SNAP25 signals were observed primarily in the IPL of BoNT/A-pretreated retinas but were absent in control and CoCl_2_-only groups. Enlarged views highlight localization within the IPL and adjacent INL. Quantitative fluorescence analyses are shown in the adjacent graphs. Scale bars: 50 μm (overview); 25 μm (insets). (**D**) Brn3a immunostaining demonstrated that BoNT/A pretreatment preserved retinal ganglion cell labeling compared with CoCl_2_-treated retinas. Scale bar: 50 μm. (**E**) TUNEL assay revealed abundant apoptotic nuclei (green) in CoCl_2_-treated retinas, which were markedly reduced in BoNT/A-pretreated samples, indicating protection against hypoxia-induced apoptosis. Scale bar: 50 μm. (**F**) IBA1 (red) and GFAP (green) were strongly upregulated in CoCl_2_-treated retinas, confirming successful induction of hypoxia, but were reduced by BoNT/A treatment, indicating suppression of glial activation. Scale bar: 50 μm. (**G**) Immunostaining for Hv1 (red) and Nox2 (green) revealed increased expression in CoCl_2_-treated retinas, which was reduced following BoNT/A pretreatment. Scale bar: 50 μm. (**H**) Western blot analyses using the same antibodies as in R28 cells showed that Nox2, Hv1, COX2, NLRP3, and TNF-α were elevated by CoCl_2_ and downregulated by BoNT/A. In contrast, SOCS3, GAP43, and Syntaxin12 were increased in BoNT/A-treated samples, suggesting enhanced anti-inflammatory and neuroprotective signaling. Statistical significance was indicated as * *p* < 0.05, ** *p* < 0.01, *** *p* < 0.001 vs. Control; # *p* < 0.05, ## *p* < 0.01, ### *p* < 0.001 vs. CoCl_2_. + *p* < 0.05, ++ *p* < 0.01, +++ *p* < 0.001 BoNT/A 0.25 IU vs. 0.5 IU. Scale bar: 50 μm.

In an ex vivo rat retinal culture model, BoNT/A treatment was applied during or after CoCl_2_ exposure to induce hypoxic stress (Figure 3A). Hematoxylin and eosin staining revealed that CoCl_2_ exposure caused progressive retinal thinning (11.4%), affecting both overall architecture and individual layers, including the RGC plus inner plexiform layer (IPL), inner nuclear layer, and outer nuclear layer (Figure 3B). At day 4, CoCl_2_-treated retinas exhibited a transient increase in thickness, likely reflecting tissue damage-related artifacts rather than genuine structural preservation. At day 8, retinas treated with 0.25 IU BoNT/A exhibited marked recovery of structural integrity, with thickness measurements comparable to control tissues (Figure 3B). At the molecular level, cleaved-SNAP25 immunofluorescence confirmed BoNT/A enzymatic activity within retinal tissue, with strong signals localized to the IPL in BoNT/A-treated groups, whereas such signals were absent in control and CoCl_2_-only retinas (Figure 3C). This enzymatic activity was associated with evidence of neuronal preservation. Brn3a staining demonstrated that CoCl_2_ treatment significantly reduced RGC labeling, whereas BoNT/A treatment restored Brn3a expression, indicating amelioration of RGC loss and recovery of neuronal integrity from 3 h to day 8 (Figure 3D). Consistently, TUNEL assays revealed a pronounced increase in apoptotic nuclei following CoCl_2_ exposure, with TUNEL-positive cells reaching 2.29%, representing a 24.4-fold increase compared to controls (0.09%). Notably BoNT/A treatment substantially reduced apoptosis, with TUNEL-positive rates decreasing to 0.15% (93% reduction) at 0.25 IU and 0.19% (92% reduction) at 0.5 IU, confirming its therapeutic efficacy in mitigating hypoxia-induced retinal cell death (Figure 3E). Beyond neuronal preservation, BoNT/A alleviated glial activation associated with hypoxic injury. CoCl_2_ exposure induced pronounced upregulation of ionized calcium-binding adapter molecule 1 and glial fibrillary acidic protein, indicative of microglial and Müller cell activation, respectively; these responses were markedly attenuated in BoNT/A-treated retinas, suggesting effective suppression of hypoxia-induced gliosis (Figure 3F). Quantitative immunofluorescence revealed changes in key oxidative regulators Hv1 and Nox2. Hv1 expression was significantly elevated in CoCl_2_-treated retinas (217.3% increase relative to controls), whereas BoNT/A treatment suppressed this upregulation by 80%, counteracting proton channel-mediated oxidative stress. Nox2 expression increased modestly (13.4%) in response to CoCl_2_; however, BoNT/A (0.25 IU and 0.5 IU) effectively reduced Nox2 levels by 48% compared to CoCl_2_ alone, further supporting its therapeutic modulation of the Nox2–Hv1 oxidative axis and alleviation of ROS-driven inflammatory signaling under hypoxic stress (Figure 3G).

Collectively, these findings demonstrate that BoNT/A functions as a therapeutic modulator that attenuates hypoxia-induced retinal damage by restoring structural integrity, mitigating apoptotic and glial responses, and suppressing oxidative stress through inhibition of the Nox2–Hv1 axis.

### 2.4. BoNT/A Modulates Inflammatory Mediators and Enhances Regenerative Protein Expression in Ex Vivo Retinal Cultures

To investigate molecular alterations further, immunoblot analyses were performed on ex vivo retinal lysates collected at 3 h, 4 days, and 8 days after CoCl_2_ exposure (Figure 3H). At 3 h, CoCl_2_ significantly increased Hv1 and tumor necrosis factor (TNF)-α compared to controls, whereas BoNT/A treatment (0.25 IU) markedly reduced their expression by approximately 0.50- and 0.67-fold, respectively, indicating an early therapeutic attenuation of inflammatory activation. Furthermore, CoCl_2_ decreased SOCS3 and Syntaxin12 levels, which were restored by BoNT/A in a dose-dependent manner, with 0.25 IU and 0.5 IU significantly enhancing SOCS3 and Syntaxin12 expression compared to CoCl_2_ alone, suggesting activation of recovery-related signaling pathways.

At 4 days, CoCl_2_ induced sustained activation of oxidative and inflammatory pathways, as evidenced by upregulation of Hv1, Nox2, NLRP3, and COX2. BoNT/A treatment (0.25 IU and 0.5 IU) effectively alleviated these pathological changes, suppressing Hv1, Nox2, and NLRP3 expression, with the higher dose additionally reducing COX2 levels. Concurrently, Growth Associated Protein 43 (GAP43), which was downregulated by CoCl_2_, was restored in a dose-dependent manner by BoNT/A, indicating promotion of regenerative and reparative processes. By 8 days, no significant differences were observed in inflammatory or regenerative protein expression across groups, suggesting that BoNT/A primarily acts during the early and intermediate phases to mitigate hypoxia-induced retinal inflammation and initiate tissue recovery.

**Figure 4:**
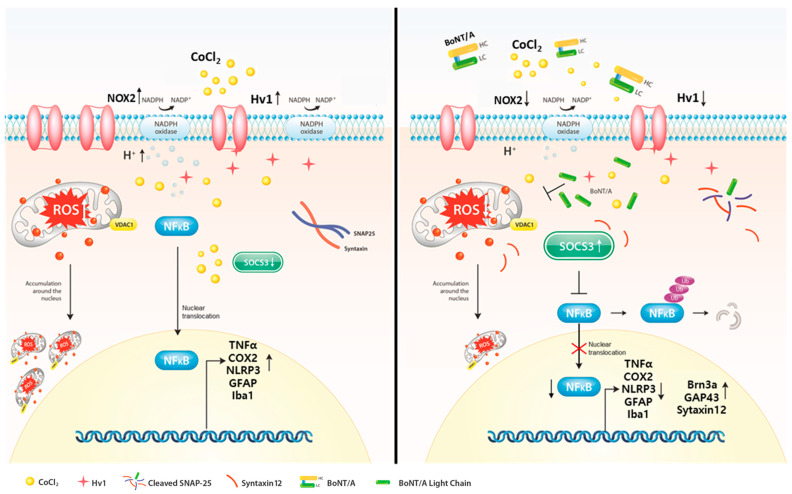
Suppression of the Nox2–Hv1 Axis and Enhancement of Neuroprotective Signaling by BoNT/A in hypoxic injury. Cobalt chloride (CoCl_2_)–induced hypoxia stabilizes HIF-1α and triggers excessive ROS production, leading to metabolic stress within cells. This condition further drives inflammation (Hv1, Nox2, NLRP3, COX2, TNF-α), retinal thinning, apoptosis, and glial activation. Pretreatment with Botulinum Toxin A (BoNT/A) suppresses the Nox2–Hv1 axis, reduces ROS and inflammatory signaling, preserves retinal ganglion cells (Brn3a), and attenuates gliosis (Iba1, GFAP). Moreover, BoNT/A enhances protective mediators (SOCS3, GAP43, Syntaxin12), thereby shifting the retinal microenvironment from a degenerative toward a neuroprotective state.

Collectively, these findings demonstrate that BoNT/A acts as a therapeutic modulator that suppresses the Nox2–Hv1 oxidative axis and alleviates ROS-driven inflammatory cascades while concurrently restoring protective mediators such as SOCS3, GAP43, and Syntaxin12. Through these mechanisms, BoNT/A promotes recovery from hypoxia-induced retinal injury and rebalances the retinal microenvironment toward a reparative and neuroprotective state (Figure 4).

## 3. Discussion

Hypoxia and inflammation are key drivers of retinal neurodegeneration in conditions such as diabetic retinopathy, glaucoma, and optic neuropathy. In experimental models, CoCl_2_ is widely used to mimic hypoxic injury by stabilizing HIF-1α, promoting ROS generation, and activating downstream inflammatory pathways [20,23,56,57]. Consistent with these mechanisms, our ex vivo retinal model demonstrated marked upregulation of Nox2, Hv1, NLRP3, COX2, and TNF-α following CoCl_2_ exposure, accompanied by retinal thinning, increased TUNEL-positive cells, and activation of microglia and Müller cells. Importantly, BoNT/A treatment alleviated these inflammatory responses and promoted the recovery of protective and regenerative proteins, including SOCS3, GAP43, and Syntaxin12, indicating its therapeutic action in mitigating hypoxia-induced retinal injury. A central mechanistic insight from our study is that BoNT/A therapeutically suppresses the Nox2–Hv1 axis, limiting ROS overproduction and subsequent activation of inflammatory cascades. Hv1, a voltage-gated proton channel, sustains Nox2 activity by providing charge compensation during ROS generation in activated microglia [19,25,58,59,60]. CoCl_2_ robustly induced Hv1 and Nox2 expression in R28 cells and ex vivo retinas, whereas BoNT/A treatment significantly downregulated their levels, thereby reducing oxidative stress and inflammatory signaling. These findings are consistent with previous studies linking Hv1 inhibition to reduced oxidative stress and neuroinflammation in central nervous system models [49,59]. By modulating this pathway after hypoxic insult, BoNT/A alleviates oxidative stress and promotes recovery of retinal structure and function.

BoNT/A activity was confirmed via cleaved-SNAP25 immunostaining, predominantly localized to the IPL, where SNAP25 is enriched in cholinergic amacrine cells [43,61]. Notably, beyond its canonical cleavage of SNAP25, our findings suggest that BoNT/A may influence Syntaxin12, another SNARE protein. Syntaxin12 underwent fragmentation in CoCl_2_-treated R28 cells, whereas its expression was restored in ex vivo retinas following BoNT/A treatment. Although classical syntaxin isoforms are established substrates of BoNT/C, these findings raise the possibility of a non-canonical modulation of Syntaxin12 by BoNT/A, which may contribute to the reparative and transport-related processes following hypoxic injury [62]. In addition to suppressing proinflammatory mediators, BoNT/A enhanced recovery-associated pathways. SOCS3, a negative regulator of JAK/STAT signaling, limits cytokine-mediated inflammation and promotes neuronal survival [63,64]. In our models, CoCl_2_ reduced SOCS3 expression, which was re-induced by BoNT/A treatment in a dose-dependent manner. Similarly, GAP43, a growth-associated protein involved in axonal regeneration and plasticity, was decreased under hypoxic conditions but significantly upregulated following BoNT/A treatment. Syntaxin12 [65], typically implicated in vesicular trafficking, was enhanced, suggesting that BoNT/A may facilitate intracellular transport and regenerative remodeling during post-injury recovery. Collectively, these findings highlight a multifaceted therapeutic role for BoNT/A in concurrently alleviating inflammation and promoting cellular restoration.

Our findings further confirmed BoNT/A-mediated restoration of retinal ganglion cell (RGC) populations. Brn3a immunostaining demonstrated preservation of RGC labeling, whereas TUNEL assays revealed reduced apoptosis in BoNT/A-treated retinas compared with CoCl_2_-exposed controls. These findings align with prior studies establishing Brn3a as a reliable marker of RGC survival and underscore the susceptibility of RGCs to hypoxic stress [66,67,68]. By mitigating RGC loss and supporting neuronal recovery, BoNT/A demonstrates therapeutic promise for retinal disorders characterized by RGC degeneration, including glaucoma and optic neuropathy.

Temporally, the most pronounced effects of BoNT/A were observed at 3 h and 4 days post-CoCl_2_ treatment, with differences diminishing by day 8. These findings suggest that BoNT/A exerts its therapeutic influence primarily during the acute and subacute phases of hypoxic injury, when modulation of the early inflammatory-to-regenerative transition is most effective. Early suppression of Nox2, Hv1, and NLRP3, alongside restoration of SOCS3 and GAP43, appears to be critical for limiting irreversible neuronal loss and initiating structural repair of retinal tissue.

Although BoNT/A was administered as a pretreatment in our experimental design, the underlying mechanisms indicate that post-hypoxia administration may also confer therapeutic benefits. Given that BoNT/A suppresses Hv1–Nox2–ROS-driven oxidative bursts and upregulates SOCS3-mediated anti-inflammatory signaling, its actions are not restricted to prophylactic use. Once hypoxic stress is established, BoNT/A could still attenuate ongoing ROS production, inhibit microglial activation, and prevent secondary neuronal injury, thereby promoting recovery of surviving retinal cells. This possibility has particular relevance for clinical scenarios where therapeutic intervention occurs after hypoxia onset, such as retinal vein occlusion or acute glaucoma attacks. Future studies are warranted to define the therapeutic window and optimal timing for BoNT/A administration following hypoxic insult.

BoNT/A did not induce detectable toxicity in either in vitro or ex vivo systems, consistent with prior studies indicating that intraocular administration at appropriate doses does not cause irreversible retinal damage [69,70,71]. Moreover, BoNT/A maintained retinal thickness and attenuated gliosis, supporting its safety and therapeutic applicability for ocular conditions associated with hypoxic stress. From a translational standpoint, these results suggest several potential clinical implications. In retinal ischemic diseases such as glaucoma, retinal vein occlusion, and diabetic retinopathy, intravitreal or periocular administration of BoNT/A may offer dual benefits-suppressing microglial-driven oxidative inflammation while preserving neuronal architecture. The well-established clinical safety of BoNT/A in ophthalmic and neurologic indications (e.g., blepharospasm, migraine, strabismus) provides a foundation for repurposing it in retinal applications, potentially as an adjunct therapy to current anti-VEGF or corticosteroid treatments. Controlled dose optimization and localized delivery approaches, such as sustained-release formulations or targeted conjugates, could further enhance its retinal bioavailability while minimizing systemic diffusion [50,72,73]. Future studies should evaluate long-term functional and electrophysiological outcomes (e.g., electroretinography, visual behavior) to confirm the sustained neuroprotective efficacy of BoNT/A in vivo. Preclinical evidence also suggests that BoNT/A may mitigate ischemic and oxidative injury in neural and ocular tissues [74], supporting its potential as a candidate for post-hypoxia intervention. Integration of BoNT/A-based therapy into multimodal treatment paradigms for retinal ischemia and glaucoma could expand its clinical utility beyond neuromuscular blockade, positioning it as a novel neuroprotective agent for hypoxia-driven retinal degeneration.

In summary, this study demonstrates that BoNT/A acts as a therapeutic modulator that alleviates hypoxia-induced oxidative stress and inflammation while promoting regenerative and reparative signaling in retinal cells. By suppressing the Nox2–Hv1–ROS axis and restoring SOCS3, GAP43, and Syntaxin12 expression, BoNT/A facilitates recovery of retinal integrity and function. Collectively, these findings position BoNT/A as a promising treatment candidate for retinal disorders characterized by neuroinflammation and hypoxia, such as retinal ischemia, glaucoma, and optic neuropathy.

## 4. Materials and Methods

### 4.1. Cell Culture, Experimental Groups, and Immunocytochemistry

R28 retinal precursor cells (a kind gift from Dr. G.M. Seigel, University of Buffalo, USA) were cultured in Dulbecco’s modified Eagle’s medium (DMEM; Thermo Fisher Scientific, Waltham, MA, USA) supplemented with 10% fetal bovine serum (FBS) and 1% penicillin/streptomycin (Sigma-Aldrich, St. Louis, MO, USA) at 37 °C in a humidified atmosphere containing 5% CO_2_. Cells were divided into three experimental groups: Control (untreated), CoCl_2_-treated (200 µM for 3 h or 24 h, Sigma-Aldrich, St. Louis, MO, USA), and CoCl_2_ + BoNT/A (0.15 IU; Botulinum neurotoxin type A, Allergan Sales, LLC, Madison, NJ, USA) group, in which cells were pretreated with BoNT/A for 2 h prior to CoCl_2_ exposure. For immunocytochemistry, R28 cells were seeded on Marigel–coated coverslips in 6-well plates (5 × 10^5^ cells/well) and treated as described above. Cells were fixed in 4% paraformaldehyde (PFA, Biosolution Co., Ltd., Suwon-si, Republic of Korea) for 15 min, permeabilized with 0.1% Triton X-100 (Sigma-Aldrich, St. Louis, MO, USA) for 3 min and blocked with 1% bovine serum albumin (BSA, Sigma-Aldrich, St. Louis, MO, USA) for 1 h. Primary antibodies against Hv1, Nox2, Brn3a, NF-κB, Syntaxin12, cleaved SNAP-25, and SOCS3 (Table 1) were applied overnight at 4 °C, followed by incubation with Alexa Fluor–conjugated secondary antibodies (1:300; Invitrogen, Carlsbad, CA, USA) for 1 h at room temperature. Nuclei were counterstained with DAPI, and coverslips were mounted using Fluorescence Mounting Medium (DAKO, Agilent Technologies, Glostrup, Denmark). Images were acquired using a confocal laser scanning microscope (LSM 880, Zeiss, Oberkochen, Germany) and slide scanner (Axio Scan.Z1; Carl Zeiss Microscopy GmbH, Jena, Germany). Fluorescence intensities were quantified in at least five randomly selected fields per group using ImageJ software (version 1.0p, National Institutes of Health, Bethesda, MD, USA) and ZEN Blue software (version 3.4, Carl Zeiss Microscopy GmbH, Jena, Germany).

### 4.2. Measurement of Mitochondrial ROS and Intracellular pH in R28 Cells

Mitochondrial ROS production and intracellular pH alterations were assessed in live R28 cells using fluorescent probes and confocal microscopy. For mitochondrial ROS measurement, cells were incubated with 5 µM MitoSOX™ Red (Thermo Fisher Scientific Inc., Waltham, MA, USA) in Hank’s Balanced Salt Solution (HBSS, Thermo Fisher Scientific, Waltham, MA, USA) for 10 min at 37 °C, washed three times, and mounted. Fluorescence images were acquired using a confocal laser scanning microscope (LSM 880, Zeiss). For live-cell measurement of intracellular pH (pHrodo™ AM Variety Pack, Thermo Fisher Scientific Inc., Waltham, MA, USA) and mitochondrial ROS (MitoSOX™ Red), cells were washed with Live Cell Imaging Solution (LCIS, Thermo Fisher Scientific Inc., Waltham, MA, USA) and incubated with a mixture containing 10 µL pHrodo™ Red, 10 µL pHrodo™ Green, 100 µL PowerLoad™, and 10 mL LCIS for 30 min at 37 °C. Following incubation, cells were washed again with LCIS, covered with 10 mL LCIS in the imaging dish, and imaged using the ImageXpress Micro Confocal (IXMC, Molecular Devices, San Jose, CA, USA) system (excitation/emission: Red, 560/585 nm; Green, 490/530 nm) at five time points taken at 20 min intervals [75,76]. For both assays, fluorescence intensity was quantified from at least five randomly selected fields per group using ImageJ (National Institutes of Health, Bethesda, MD, USA) and ZEN Blue software (version 3.4; Carl Zeiss Microscopy GmbH, Jena, Germany).

### 4.3. Retina Explant Preparation and Organotypic Culture

Sprague–Dawley (SD) rats (8 weeks old, Orient Bio Inc., Seongnam, Republic of Korea) were used for ex vivo retinal explant culture, and all animal procedures were approved by the Institutional Animal Care and Use Committee of Bundang CHA Medical Center (Approval Code: IACUC250106, Approval Date: 1 June 2025) were used for ex vivo retinal explant culture. A total of 36 rats were used (*n* = 3 per experimental group: Control, CoCl_2_, CoCl_2_ + BoNT/A 0.25 IU, and CoCl_2_ + BoNT/A 0.5 IU) at each of three sampling time point (3 h, 4 days, 8 days). Both eyes (OD and OS) from each rat were enucleated immediately after euthanasia and processed pairwise to ensure consistency between biological replicates. Thus, 6 retinas were prepared per group per time point, and the contralateral eye was used to confirm reproducibility between right and left retinal explants. Retinas were dissected in Neurobasal-A medium (Thermo Fisher Scientific Inc., Waltham, MA, USA) and placed onto Millicell culture inserts (Millipore; 0.4-µm pore diameter, Merck Millipore Ltd., Burlington, MA, USA) with the ganglion cell layer (GCL) facing upward. Inserts were placed in 6-well plates and maintained at 37 °C in a humidified 5% CO_2_ incubator. Explants were cultured for up to 8 days in Neurobasal-A medium supplemented with 0.8 mM L-glutamine, 2% B27, 1% N2 (Thermo Fisher Scientific), and 2% penicillin/streptomycin (Sigma-Aldrich). Media were completely replaced on days 0, 1, and 3, and subsequently half-replaced every two days. After a 24 h stabilization period, hypoxic stress was induced by CoCl_2_ (300 µM) treatment. For treatment groups, explants were treated with BoNT/A (0.25 or 0.5 IU) for 2 h prior to CoCl_2_ exposure (300 µM for 48 h—incubation). Sampling was conducted at 3 h, 4 days, and 8 days post-treatment. At each time point, individual retinas were bisected along the vertical meridian to obtain matched tissue halves for histological/immunofluorescence and Western blot analyses, respectively.

Inclusion and Exclusion Criteria:

Only healthy rats without ocular abnormalities were included in the study. Animals exhibiting corneal opacity, ocular infection, or mechanical damage during dissection were excluded; however, no animals were excluded in this study. All explants were morphologically intact, and both eyes were successfully processed for paired analyses.

### 4.4. Western Blotting

R28 cells and retinal explants were lysed in PRO-PREP buffer (iNtRON Biotechnology, Seongnam-si, Republic of Korea). Protein concentrations were determined using the BCA method (Thermo Fisher Scientific Inc., Waltham, MA, USA). Equal amounts of protein (20–30 µg) were separated by SDS-PAGE and transferred to PVDF membranes (GE Healthcare, Chicago, IL, USA). Membranes were blocked with 5% skim milk in TBS-T for 1 h at room temperature and incubated overnight at 4 °C with primary antibodies (Table 1) against HIF-1α, Hv1, Nox2, NLRP3, COX2, NF-κB, SOCS3, GAP43, and Syntaxin12. After washing, membranes were incubated with horseradish peroxidase (HRP)-conjugated secondary antibodies (1:5000; Jackson ImmunoResearch, West Grove, PA, USA or GeneTex, Irvine, CA, USA) for o/n at 4 °C. Protein bands were visualized using enhanced chemiluminescence solutions (Bio-Rad Laboratories, Hercules, CA, USA) and detected using an ImageQuant™ LAS 4000 imaging system (GE Healthcare Life Sciences, Uppsala, Sweden). Band intensities were quantified with ImageJ software (National Institutes of Health, Bethesda, MD, USA), and β-actin was used as the loading control.

### 4.5. TUNEL Assay

Apoptotic cells were detected using the In Situ Cell Death Detection Kit (Roche, Basel, Switzerland) in both retinal sections and cultured R28 cells.

Retinal sections: Paraffin-embedded retinal sections (5 µm) were deparaffinized, permeabilized with proteinase K (20 µg/mL, Thermo Fisher Scientific Inc., Waltham, MA, USA), and incubated with the TUNEL reaction mixture for 1 h at 37 °C in a humidified chamber. After washing, nuclei were counterstained with DAPI.

R28 cells: Cells were seeded on poly-D-lysine (Sigma-Aldrich Co. LLC., St. Louis, MO, USA)—coated coverslips, treated under the indicated experimental conditions, and fixed with 4% paraformaldehyde for 15 min. After permeabilization with 0.1% Triton X-100 in 0.1% sodium citrate for 2 min on ice, cells were incubated with the TUNEL reaction mixture for 1 h at 37 °C, followed by DAPI nuclear counterstaining. For both tissue and cell preparations, slides/coverslips were mounted with Fluorescence mounting medium (DAKO, Glostrup, Denmark) and imaged using a Zeiss LSM 880 confocal microscope (Zeiss). The proportion of TUNEL-positive nuclei was quantified in at least five randomly selected fields per sample using ImageJ (National Institutes of Health, Bethesda, MD, USA) and ZEN Blue software (version 3.4; Carl Zeiss Microscopy GmbH, Jena, Germany).

### 4.6. Histology and Ex Vivo Retina Immunofluorescence

For histological analysis, retinal explants were fixed in 4% paraformaldehyde (PFA) overnight, dehydrated, cleared, and embedded in paraffin. Sections (5 µm) were cut, mounted, and stained with hematoxylin and eosin (H&E). Images were acquired using a slide scanner (Axio Scan.Z1). Retinal layer thicknesses, including the inner plexiform layer (IPL), inner nuclear layer (INL), and outer nuclear layer (ONL), were measured in five randomly selected fields per retina using and ZEN Blue software (version 3.4).

For immunofluorescence analysis, two types of tissue preparation were used depending on the target proteins.

Cryosection-based immunofluorescence: Retinal explants were cryosectioned at 14 µm thickness, permeabilized with 0.1% Triton X-100, and blocked with 1% bovine serum albumin (BSA) for 1 h. Sections were incubated overnight at 4 °C with a primary antibody against cleaved SNAP-25 (Table 1). After washing, sections were incubated with Alexa Fluor–conjugated secondary antibodies (1:300; Thermo Fisher Scientific) for 1 h at room temperature, counterstained with DAPI, and mounted with Fluorescence Mounting Medium.

Paraffin section-based immunofluorescence: Paraffin-embedded retinal sections (5 µm) were deparaffinized, rehydrated, subjected to antigen retrieval, and permeabilized with 0.1% Triton X-100. After blocking with 1% BSA, sections were incubated overnight at 4 °C with primary antibodies against Brn3a, Hv1, Nox2, IBA1, and GFAP (Table 1). Alexa Fluor–conjugated secondary antibodies (1:300) were applied for 1 h at room temperature, followed by DAPI counterstaining and mounting with Fluorescence Mounting Medium. Images for both cryosection and paraffin-based staining were acquired using a Zeiss LSM 880 confocal microscope (Zeiss), and fluorescence intensities were quantified with ImageJ and ZEN Blue software (version 3.4).

### 4.7. Quantification and Statistical Analysis

All results were presented as mean ± standard error of the mean (SEM). Statistical analyses were performed using GraphPad Prism 9 software (GraphPad Software, La Jolla, CA, USA). One-way analysis of variance (ANOVA) with Tukey’s post hoc test was used for normally distributed data, while the Mann–Whitney U test was applied to non-parametric data. Statistical significance was defined as *p* < 0.05. Details of statistical criteria are described in the figure legends.

## 5. Conclusions

We demonstrate that BoNT/A exerts robust protective effects in R28 retinal precursor cells and ex vivo rat retinal explants subjected to cobalt chloride–induced hypoxic stress. BoNT/A attenuated oxidative stress by suppressing the Nox2–Hv1–ROS axis, reduced apoptotic cell death, and limited glial activation. Simultaneously, it enhanced the expression of protective and regenerative mediators, including SOCS3, GAP43, and Syntaxin12, promoting neuronal survival and preserving retinal architecture. Mechanistically, BoNT/A’s enzymatic activity was confirmed via cleaved-SNAP25 immunostaining in the IPL, with additional evidence suggesting potential non-canonical effects on Syntaxin12. Notably, BoNT/A preserved RGC integrity, maintained retinal layer thickness, and mitigated hypoxia-induced apoptosis, emphasizing its dual role in anti-inflammatory modulation and neuroprotection.

These findings position BoNT/A as a promising therapeutic candidate for retinal disorders characterized by hypoxia and neuroinflammation, including retinal ischemia, glaucoma, and optic neuropathy. By simultaneously dampening inflammatory cascades and enhancing regenerative signaling, BoNT/A offers a potential strategy to protect retinal neurons and preserve visual function under hypoxic conditions. To further strengthen its translational relevance, future studies should evaluate the long-term functional efficacy of BoNT/A using in vivo ischemic retinal models, including electrophysiological and behavioral assessments. Additionally, optimization of dosage, delivery route, and treatment timing will be essential to ensure therapeutic safety and effectiveness in clinical applications. Collectively, these recommendations will help advance BoNT/A toward its potential use as a targeted neuroprotective therapy for hypoxia-related retinal diseases.

## Figures and Tables

**Table 1:** Primary and secondary antibodys used for Western blot and Histological analysis.

Antibody	Host Species	Company	Catalog No.	Dilution and Application
Hv1	Rabbit	LSBio, Seattle, WA, USA	LS-B5319	1:200 (ICC, IF), 1:1000 (WB)
Nox2	Mouse	Invitrogen, Carlsbad, CA, USA	MA5-18052	1:200 (ICC, IF), 1:1000 (WB)
Brn3a	Mouse	Millipore, Burlington, MA, USA	MAB1585	1:50 (ICC), 1:1000 (WB)
Brn3a	Rabbit	Abcam, Cambridge, UK	ab245230	1:200 (IF)
NF-κB (p65)	Rabbit	Cell Signaling Technology, Danvers, MA, USA	8242S	1:200 (ICC)
p-NFκB p65	Mouse	Cell Signaling Technology, Danvers, MA, USA	3036S	1:1000 (WB)
Syntaxin12	Rabbit	Proteintech, Rosemont, IL, USA	14259-1-AP	1:200 (ICC), 1:1000 (WB)
VDAC1	Rabbit	Abcam, Cambridge, UK	ab15895	1:200 (ICC)
SNAP-25 BoTox-A cleaved	Mouse	MyBioSource, San Diego, CA, USA	MBS350064	1:50 (ICC, IF)
SOCS3	Rabbit	Invitrogen, Carlsbad, CA, USA	PA5-87485	1:200 (IF)
COX-2	Rabbit	Cell Signaling Technology, Danvers, MA, USA	12282S	1:1000 (WB)
NLRP3	Mouse	Proteintech, Rosemont, IL, USA	NBP2-12446	1:1000 (WB)
TNF-α	Rabbit	GeneTex, Irvine, CA, USA	GTX110520	1:1000 (WB)
GAP43	Rabbit	Abcam, Cambridge, UK	ab75810	1:1000 (WB)
GFAP	Mouse	Cell Signaling Technology, Danvers, MA, USA	3670S	1:300 (IF)
Iba1	Rabbit	Cell Signaling Technology, Danvers, MA, USA	17198S	1:300 (IF)
HIF-1α	Rabbit	Invitrogen, Carlsbad, CA, USA,	PA1-16601	1:1000 (WB)
iNOS	Rabbit	Invitrogen, Carlsbad, CA, USA,	PA3-030A	1:1000 (WB)
VEGF	Rabbit	GeneTex, Irvine, CA, USA	GTX102643	1:1000 (WB)
β-Actin	Mouse	Santa Cruz Biotechnology, Dallas, TX, USA	SC-47778	1:5000 (WB), Loading control
Mouse IgG(H+L) Alexa Fluor 488	Donkey	Invitrogen, Carlsbad, CA, USA	A32766	1:300 (ICC, IF)
Mouse IgG(H+L) Alexa Fluor 555	Donkey	Invitrogen, Carlsbad, CA, USA	A32773	1:300 (ICC, IF)
Rabbit IgG(H+L) Alexa Fluor 488	Donkey	Invitrogen, Carlsbad, CA, USA	A32790	1:300 (ICC, IF)
Rabbit IgG(H+L) Alexa Fluor 555	Donkey	Invitrogen, Carlsbad, CA, USA	A32794	1:300 (ICC, IF)

## Data Availability

The data that support the findings of this study are available from the corresponding author upon reasonable request.

## Supplementary Materials

The following supporting information can be downloaded at: https://www.mdpi.com/article/10.3390/ijms262110806/s1.

## Abbreviations

AMD	Age-related macular degeneration
BRB	Blood–retinal barrier
BoNT/A	Botulinum neurotoxin type A
CNS	Central nervous system
CoCl_2_	Cobalt chloride
DR	Diabetic retinopathy
ER	Endoplasmic reticulum
GFAP	Glial fibrillary acidic protein
HIF-1α	Hypoxia-inducible factor 1-alpha
Hv1	Voltage-gated proton channel (HVCN1)
IBA1	Ionized calcium-binding adaptor molecule 1
INL	Inner nuclear layer
IPL	Inner plexiform layer
LPS	Lipopolysaccharide
NF-κB	Nuclear factor kappa-light-chain-enhancer of activated B cells
NLRP3	NOD-, LRR- and pyrin domain-containing protein 3 inflammasome
Nox2	NADPH oxidase 2
ONL	Outer nuclear layer
OPL	Outer plexiform layer
OS	Outer segments
RGC	Retinal ganglion cell
ROS	Reactive oxygen species
SNAP-25	Synaptosomal-associated protein 25 kDa
SOCS3	Suppressor of cytokine signaling 3
VDAC1	Voltage-dependent anion channel 1
