# Supplementary material for: Therapeutic Modulation of the Nox2–Hv1–ROS Axis by Botulinum Neurotoxin A Confers Protection Against CoCl2-Induced Retinal Hypoxic Injury
Source: Int J Mol Sci. 2025 Nov 6;26(21):10806. doi: 10.3390/ijms262110806 (PMC12608730; doi:10.3390/ijms262110806)
Supplement: Supplementary file 1 [file ijms-26-10806-s001.zip › ijms-3951303-supplementary.pdf]

Supplementary Table S1. Quantitative summary of cell viability in R28 retinal precursor cells following $CoCl_2$-induced hypoxic stress and BoNT/A treatment. Values represent mean ± SD from three independent experiments (n=6, n=4). Statistical analysis was performed using one-way ANOVA followed by Tukey's post hoc test.

Group	Cell Viability (%) ± SD	Mean± SD	n	p-value(vs. $CoCl_2$)
Control	100 ± 4.8	1.000±0.048	6	0.0043
$CoCl_2$	85.3 ± 8.8	0.853±0.088	6	-
$CoCl_2$ + BoNT 0.07 IU	90.2 ± 3.6	0.902±0.036	4	N.S
$CoCl_2$ + BoNT 0.15 IU	94.6 ± 3.0	0.946±0.030	4	0.0429
$CoCl_2$ + BoNT 0.3 IU	92.7 ± 1.8	0.927±0.018	4	N.S

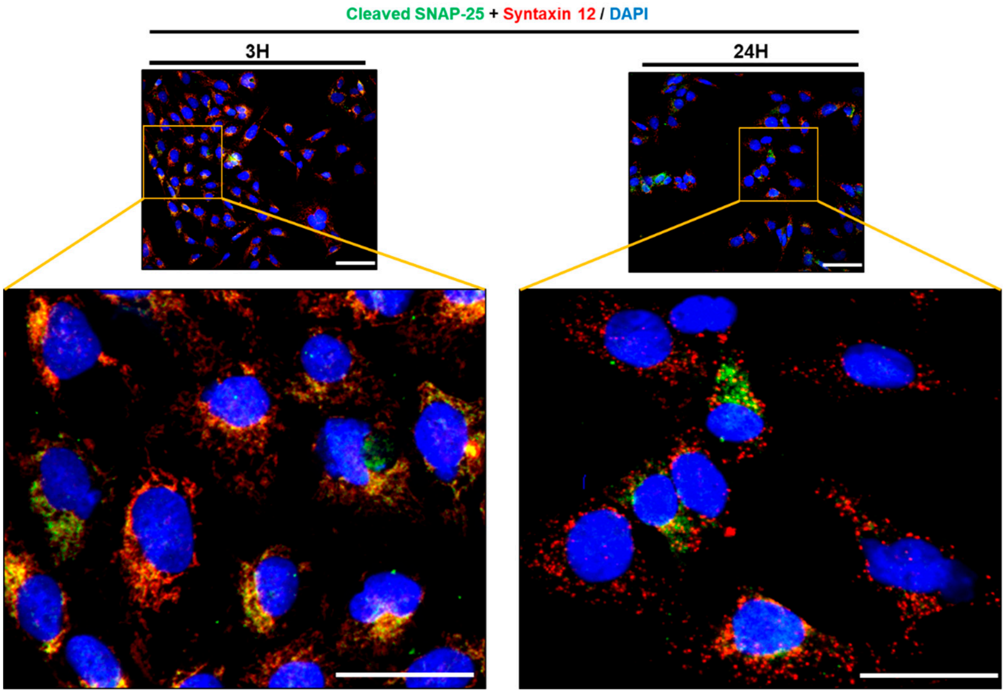

Supplementary Figure S1. Dual immunostaining of cleaved-SNAP25 (green) and Syntaxin12 (red) demonstrated temporal patterns at 3 h and 24 h. Syntaxin12 appeared elongated at 3 h but fragmented at 24 h. The concurrent appearance of cleaved-SNAP25 and fragmented Syntaxin12 suggests that BoNT/A may cleave both SNAP25 and Syntaxin12 in R28 cells. Scale bars: 50 µm (overview); 25 µm (magnified insets).

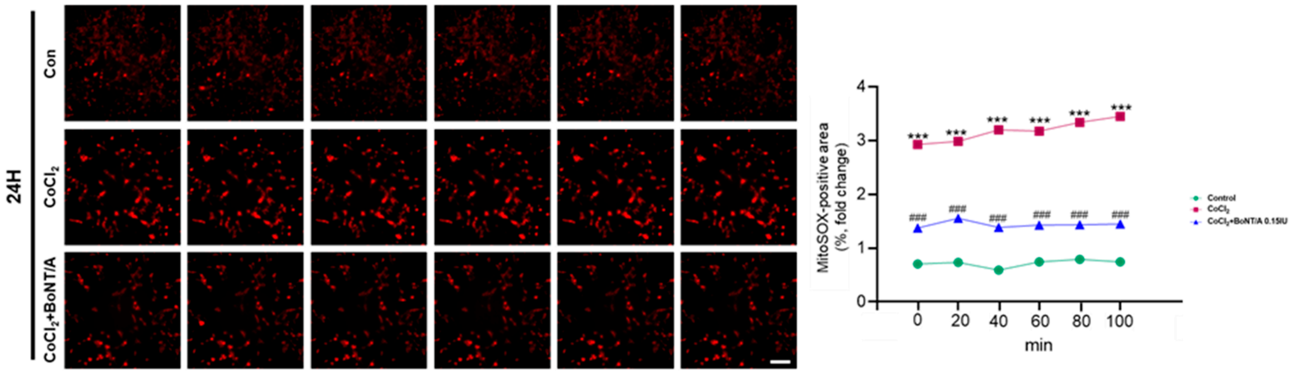

Supplementary Figure S2. Live-cell imaging of mitochondrial ROS production with MitoSOX staining, captured every 20 min across six time points, demonstrating dynamic changes under control, $CoCl_2$, and BoNT/A + $CoCl_2$ conditions. Scale bars: 200 µm
